# Synergistic Enhancement of Catalytic Activities in Ligand‐Regulated Carbon Dots‐Ferric Ion Nanozymes via UV‐Enhanced Peroxidase‐Oxidase

**DOI:** 10.1002/advs.202519424

**Published:** 2026-02-28

**Authors:** Xiangli Li, Jiaxin Dong, Dechang Jia, Yu Zhou, Baoqiang Li

**Affiliations:** ^1^ Institute for Advanced Ceramics State Key Laboratory of Urban Water Resource and Environment Harbin Institute of Technology Harbin P. R. China

**Keywords:** CDs‐Fe, ligand‐regulated CDs, nanozyme, synergistic catalysis, UV‐enhanced peroxidase‐oxidase

## Abstract

Species of metal ions in carbon dots‐metal ions complex nanozymes boost enzyme‐mimicking activities. However, the relationship between ligand regulation of the catalytic activity of carbon dots‐metal ions complex nanozymes still lacks clarity, which hinders the design of high‐performance nanozymes. Herein, to understand the influence of ligands on nanozyme activities, three kinds of carbon dots‐ferriic ions (CDs‐Fe) nanozymes with three ligands, including allyltriphenylphosphonium bromide (TPP), 1‐hydroxyethane‐1,1‐diphosphonic acid (HEDP), and diethylenetriaminepentaacetic acid (DTPA), are synthesized via sequential hydrothermal carbonization and coordination chemistry. The effects of ligand on peroxidase (POD)‐mimicking and UV‐induced oxidase‐mimicking activities are then systematically evaluated. All CDs‐Fe nanozymes exhibit dual POD‐mimicking and UV‐induced oxidase (OXD)‐mimicking activities. Type of ligand significantly determines catalytic performance, for example, HEDPCDs‐Fe exhibits the highest POD‐mimicking activity (V_m_ 2.68 × 10^−7^ M s^−1^), meanwhile TPPCDs‐Fe showing superior UV‐induced OXD‐mimicking activity (V_m_ 2.26 × 10^−7^ M s^−1^). Crucially, the synergistic effect of UV‐enhanced POD‐OXD is observed, where UV‐induced processes enhanced POD‐mimicking catalysis, motivating significantly improved reaction kinetics with lower K_m_ (1.05 mm) and higher V_m_ (5.17 × 10^−7^ M s^−1^). POD‐mimicking activity with UV irradiation is 1.93 times that in the dark. Ligand‐regulation strategy of CDs‐Fe nanozyme based on coordination chemistry offers an idea to design and synthesize high catalytic activities of CDs‐metal ions nanozymes.

## Graphical abstract

Three CDs‐Fe nanozymes functionalized with tailor‐made ligands (TPP, HEDP, and DTPA) are synthesized via hydrothermal carbonization and coordination chemistry. A ligand‐regulation strategy for CDs‐Fe nanozymes achieves tunable enzyme‐mimicking activities and a powerful synergistic effect between POD and UV‐induced OXD pathways, paving the way for designing high‐performance nanozymes.

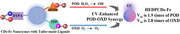

## 1 Introduction

Carbon dots (CDs) have emerged as versatile nanozymes due to their tunable physicochemical properties, excellent biocompatibility, and low cytotoxicity, which makes them promising alternatives to natural enzymes in catalysis, biosensing, and therapeutics [1, 2, 3, 4, 5]. The catalytic activity of CD‐based nanozymes is highly dependent on structural features such as heteroatom doping, surface functional moieties, and morphological engineering [6, 7, 8, 9, 10]. For instance, N/P/S doping modulates the electron density of CD surfaces, thereby enhancing peroxidase (POD)‐mimicking or oxidase (OXD)‐mimicking activities through abundant active sites [8, 11, 12, 13]. Carboxyl, amino, or hydroxyl groups further tailor the binding affinity toward substrates (H_2_O_2_, 3,3',5,5'‐tetramethylbenzidine (TMB)) and influence the catalytic kinetics [14, 15, 16]. However, most studies have focused on single‐factor modulation (sole doping or surface modification, etc.) [17], a comprehensive understanding of how CDs moieties (e.g., ligand‐functionalized vs. non‐functionalized) synergistically govern multiple enzyme‐mimicking activities is still lacking.

The incorporation of metal ions (e.g., Fe^3+^, Cu^2+^, Co^2+^) into CDs to form CD‐metal complexes has advanced nanozyme performance, as metal ions act as additional active centers and modulate the electronic structure of CDs [18, 19, 20, 21, 22, 23]. For example, Fe‐doped CDs exhibit multi‐enzyme activities for phenolic compounds detection [24, 25]. Cu^2+^‐modified graphene oxide‐CDs with dual POD‐mimicking/NADH POD‐mimicking activities, where Cu^2+^ coordinated with surface carboxyl groups of CDs to facilitate H_2_O_2_ activation [26, 27]. More recently, EDTA‐functionalized CDs‐metal chelates (CDs_EDTA_‐Me) demonstrated that metal ion species determined the enzyme‐mimicking type [16, 28, 29, 30]. Bimetallic systems, such as Cu/Mn–N_4_ co‐doped CDs, further emulate natural SOD active sites, demonstrating broad‐spectrum antioxidant activity against multiple reactive oxygen species (ROS) [31, 32, 33]. Collectively, these studies confirm that metal doping can create diverse and efficient catalytic sites within CDs, thereby expanding their potential applications in biomedicine and sensing.

Despite these advances, most prior CD‐metal nanozyme studies rely on non‐specific coordination between CDs’ surface functional groups (‐NH_2_, ‐COOH) and metal ions, resulting in ambiguous coordination structures [34, 35, 36, 37]. Additionally, dual‐enzyme activities in existing nanozymes are typically independent without synergistic enhancement, especially under photo irradiation [36, 37]. Therefore, the precise control over the metal coordination environment in CDs, particularly via precursor ligands, remains underexplored [38, 39, 40, 41, 42, 43, 44], and integrating POD‐mimicking behavior with UV‐induced OXD‐mimicking properties has rarely been achieved [45].

Herein, we report a series of functionalized ferric ions‐chelated carbon dots (CDs‐Fe) nanozymes using tailor‐made ligands incorporated during the precursor stage. These CDs‐Fe nanozymes were designed to possess dual POD‐mimicking and UV‐induced OXD‐mimicking activities, where the ligand type (allyltriphenylphosphonium bromide, TPP; 1‐hydroxyethane‐1,1‐diphosphonic acid, HEDP; diethylenetriaminepentaacetic acid, DTPA) is tailor‐made to regulate catalytic performance. This ligand strategy enables precise modulation of Fe^3+^ coordination environments. CDs‐Fe nanozymes with tailor‐made ligands simultaneously exhibit dual catalytic activities: POD‐mimicking activity and UV‐induced OXD‐mimicking activity. Moreover, these two activities exhibit a synergistic enhancement: UV‐generated ROS promote H_2_O_2_ decomposition in POD‐mimicking reactions, while POD‐mediated H_2_O_2_ activation enhances UV‐induced O_2_ reduction. This work provides a novel strategy for designing high‐performance CD‐based nanozymes via ligand regulation.

## 2 Results and Discussion

### 2.1 Synthesis of CDs‐Fe Nanozymes with Tailor‐Made Ligands Based on Coordination Chemistry

**SCHEME 1:**
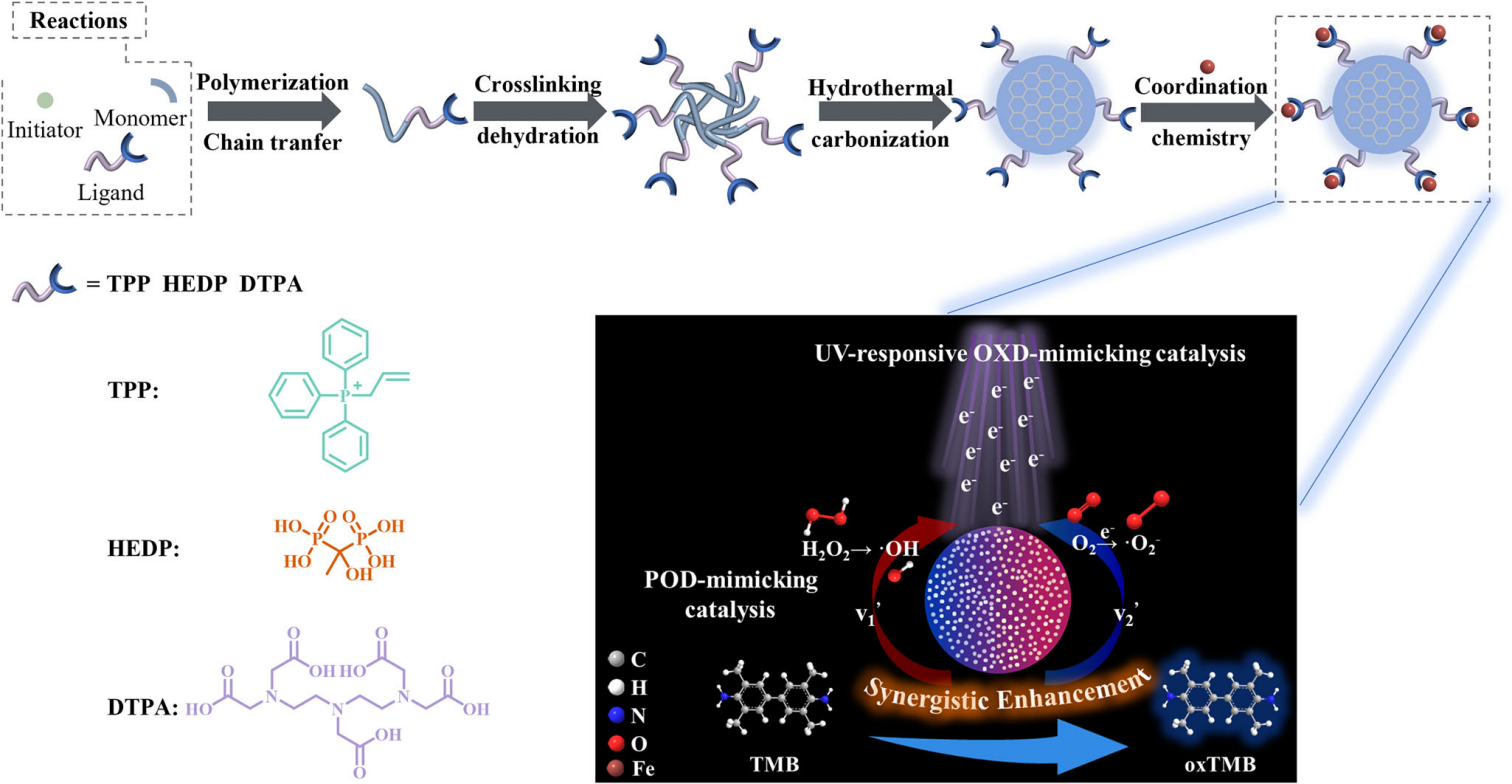
Schematic illustration for synthesizing CDs‐Fe nanozyme and synergistic enhancement of UV‐enhanced POD‐OXD‐mimicking activities in CDs‐Fe nanozymes by UV irradiation.

Toward developing highly active nanozymes, this study employed a rational design strategy centered on tuning the electronic and steric properties of iron centers in CDs through tailor‐made ligands. First, three ligands (TPP, HEDP, DTPA) with different coordinating atoms (N, O, P) and spatial structures were selected and underwent hydrothermal carbonization with acrylamide (AM) to yield CDs with defined surface functionalities [46]. As illustrated in Scheme 1, the synthetic pathway is proposed to involve the initial free‐radical polymerization of AM, followed by ligand grafting via a chain‐transfer reaction, and subsequent cross‐linking and hydrothermal carbonization [47, 48, 49]. Three distinct types of CDs‐Fe nanozymes with varied local coordination environments were then constructed via coordination with Fe^3+^ (Figure S1). Each CDs‐Fe nanozyme type possesses a unique local coordination environment, which is designed to differentially modulate the iron center's reactivity and its UV‐enhanced POD‐OXD‐mimicking activity.

These ligands were selected based on their distinct coordination chemistry and potential to tailor Fe^3+^‐mediated catalytic activities. TPP represents a class of phenylphosphine ligands that coordinate with metal ions primarily through the P atom. HEDP belongs to a category of phosphonate ligands that coordinate with metal ions via P‐O bonds. DTPA is a representative carboxylate‐amine ligand that coordinates with metal ions through N and O atoms. In addition, studies have shown the extensive application of these ligands in chelating metal ions, confirming that their selection is based on prior research and coverage of core ligand functionalities [50, 51, 52]. The coordination of TPP with Fe^3+^ is achieved via phosphorus, and its bulky triphenyl moiety introduces steric hindrance, while moderately modulating Fe^3+^ electron density, favorable for UV‐induced charge transfer. HEDP's dense O‐P‐O groups strongly facilitate electron transfer from the CDs core, accelerating the Fe^2+^/Fe^3+^ redox cycle and thereby enhancing POD‐mimicking activity. Additionally, TPP, HEDP, and DTPA as ligands for synthesizing CD‐Fe nanozymes help elucidate the influence of different coordination atoms with ferric ions (P‐Fe, O‐Fe, N‐Fe) on their catalytic activity. DTPA's hexadentate N/O coordination forms rigid chelates, stabilizing Fe^3+^ to enhance structural robustness. Literature coordination constants (log K: 21.0 for HEDP, 28.6 for DTPA) confirm their binding affinity [53, 54]. This selection enables systematic investigation of how coordinating atoms, steric/electronic effects, and coordination of ligands influence nanozyme performance in CDs‐Fe nanozymes.

### 2.2 Quantitative Characterization of Coordination Ability of CDs with Tailor‐Made Ligands for Ferric Ions

**FIGURE 1:**
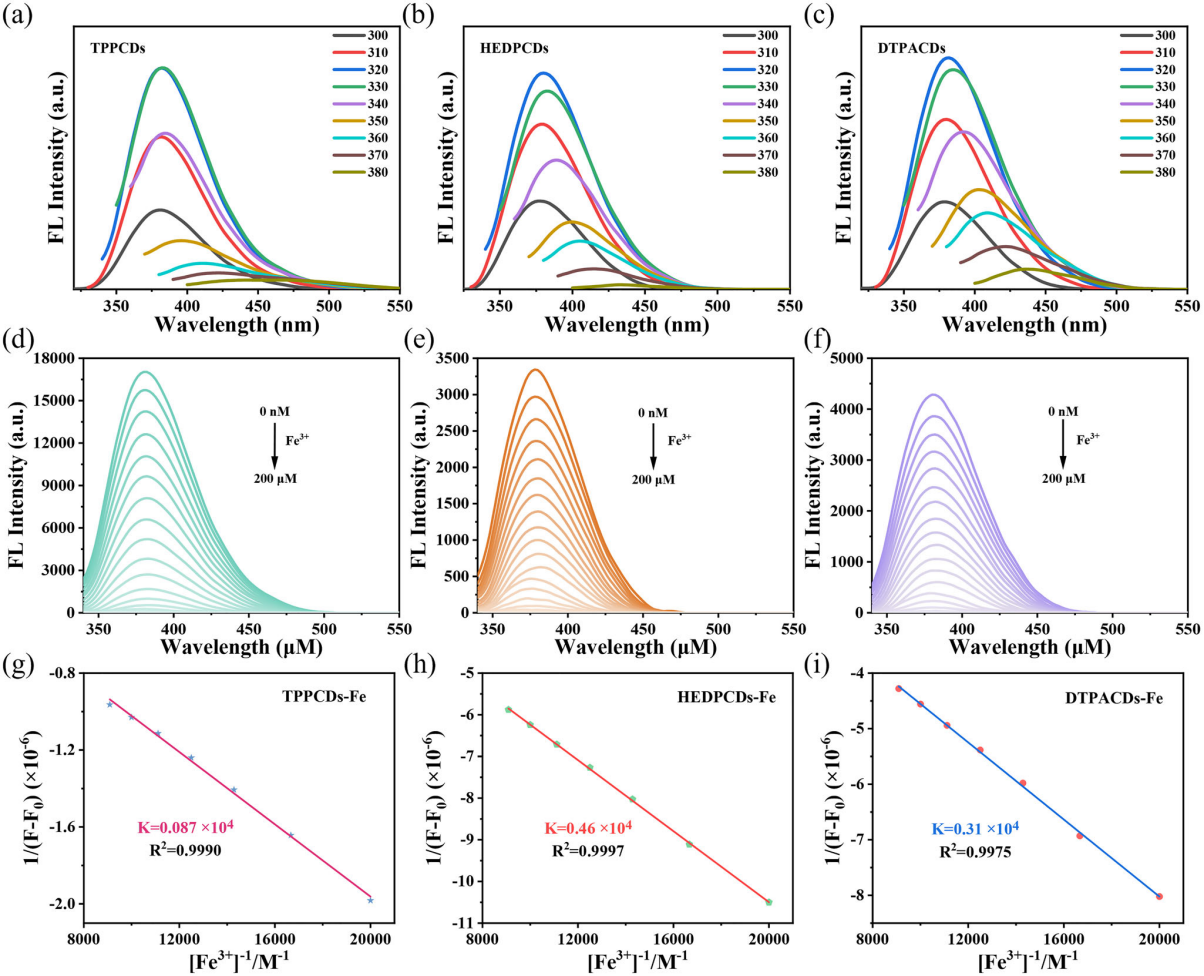
The Fe^3+^ coordination ability of CDs with tailor‐made ligands (TPP, HEDP, DTPA). (a–c) Fluorescence spectra of (a) TPPCDs, (b) HEDPCDs, and (c) DTPACDs. (d–f) Fluorescence intensity of (d) TPPCDs, (e) HEDPCDs, and (f) DTPACDs at various Fe^3+^ concentrations. (g–i) The coordination constants of (g) TPPCDs, (h) HEDPCDs, and (i) DTPACDs for Fe^3+^.

The optical characteristics of CDs with tailor‐made ligands and CDs‐Fe nanozymes were characterized to demonstrate the coordination ability of CDs. The fluorescence spectra of TPPCDs, HEDPCDs, and DTPACDs (Figure 1a–c) display excitation‐dependent behavior, with optimal excitation and emission wavelengths of 320 and 380 nm, respectively. As depicted in Figure 1d–f, the fluorescence intensity of CDs with tailor‐made ligands gradually quenched with the increase of Fe^3+^ concentration. This phenomenon is attributed to static quenching resulting from coordination between Fe^3+^ and CDs with tailor‐made ligands, leading to static fluorescence quenching [55]. Furthermore, the coordination constant (K) values for Fe^3+^ with TPPCDs, HEDPCDs, and DTPACDs are 0.087 × 10^4^ M^−1^, 0.46 × 10^4^ M^−1^, and 0.31 × 10^4^ M^−1^, respectively. (Figure 1g–i). These coordination constant values confirm the formation of complexes between Fe^3+^ and TPPCDs, HEDPCDs, as well as DTPACDs.

Figure S2 presents the UV–vis absorption and fluorescence emission spectra of three ligand‐functionalized CDs (TPP‐, HEDP‐, and DTPA‐CDs) and their Fe‐coordinated derivatives. Fe coordination induces distinct changes in UV absorbance and marked fluorescence quenching. This phenomenon stems from the coordination interaction between Fe^3+^ and the ligands on the CDs, collectively providing spectroscopic evidence for successful Fe binding in each platform. The distinct differences observed in the UV–vis absorption spectra of the three types of CDs with tailor‐made ligands (TPP, HEDP, and DTPA) (Figure S2a–c) are attributed to the effective modulation of their electronic properties by the precursor structures. The enhanced absorption of TPPCDs at approximately 240 nm is ascribed to enhanced conjugation and p‐π interactions induced by the allyl group and phosphorus atom. The *π–π* ^*^ transition peak at 270 nm is attributed to TPP's conjugated aromatic rings, confirming abundant π‐electrons. HEDPCDs exhibited intermediate absorption peak positions and intensities, resulting from modulation of the energy gap by phosphonyl and hydroxyl groups. In contrast, DTPACDs showed stronger and broader short‐wavelength absorption, which was mainly caused by the n‐π* transitions from carboxyl and amine groups. These spectral variations confirmed that the conjugation degree, electron density, and bandgap of the CDs are modulated by the specific surface functional ligands, indicating successful precursor modification.

### 2.3 Qualitative Characterization of Coordination Interactions Between HEDPCDs and Ferric Ions

**FIGURE 2:**
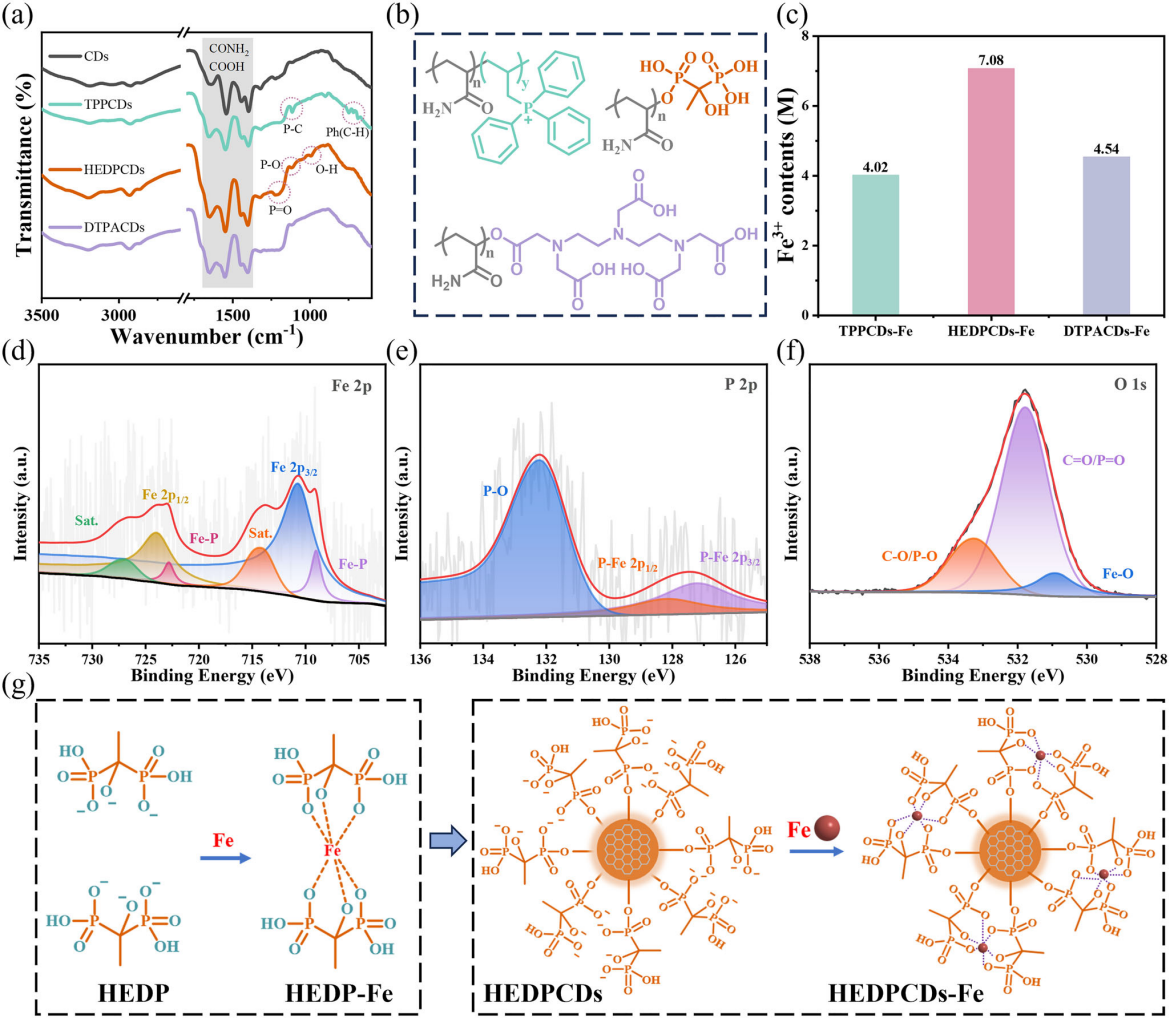
TPP, HEDP, and DTPA ligands of CDs moieties and the coordination structure of HEDPCDs‐Fe: absorbance, fluorescence, FTIR, XPS, and coordination structure. (a) FTIR spectra of TPPCDs, HEDPCDs, and DTPACDs. (b) Schematic diagram of the structure of TPPCDs, HEDPCDs, and DTPACDs. (c) The Fe^3+^ content in CDs‐Fe with tailor‐made ligands. (d–f) XPS high‐resolution scans of (d) Fe 2p, (e) P 2p, and (f) O 1s of HEDPCDs‐Fe. (g) Diagram of HEDPCDs‐Fe coordination structure inferred from HEDP‐Me coordination structure.

Using HEDPCDs as a representative example, the structures of both HEDPCDs and HEDPCDs‐Fe were qualitatively characterized. Figure 2a presents the FTIR spectra of the unmodified CDs and CDs with tailor‐made ligands (TPP, HEDP, DTPA). In contrast to CDs derived solely from AM, the CDs with tailor‐made ligands display distinct absorption features characteristic of their respective precursor ligands. For TPPCDs, C‐H bending vibrations of monosubstituted benzene rings are observed at 748, 718, and 689 cm^−1^, confirming preservation of the aromatic structure from the TPP moiety. A peak near 1115 cm^−1^, attributed to the P‐Ph vibration, further verifies the successful incorporation of the TPP moiety. The HEDPCDs spectrum shows a strong broad band near 1224 cm^−1^, corresponding to the P═O stretching vibration of the phosphonate moiety. Additional peaks between 1117 and 992 cm^−1^ are assigned to P‐O and O‐H vibrations, collectively indicating the successful grafting of HEDP ligand onto the CDs surface. For DTPACDs, the characteristic doublet of the carboxylate ion (‐COO^−^), arising from C═O and C–N stretching, overlaps with the amide absorptions from AM (C═O and C‐O stretching) at 1653 and 1403 cm^−1^. These spectral results confirm the successful preparation of three types of CDs with different tailor‐made ligands via the precursor‐based strategy (Figure 2b). The TEM image reveals HEDPCDs and HEDPCDs‐Fe as uniformly sized nanoparticles with excellent dispersibility; no significant aggregation is observed (Figure S3a). HEDPCDs‐Fe maintains well‐preserved dispersibility, with no obvious particle growth or aggregation (Figure S3b). This observation directly demonstrates that Fe coordination does not disrupt the original nanomorphology of ligand‐functionalized CDs. The spatial distribution of C and Fe, as revealed by EDS mapping, confirms the successful modification of HEDPCDs with Fe^3+^ (Figure S3c–e).

The ICP‐MS analysis (Figure 2c) shows HEDPCDs (7.08 × 10^−4^ M) exhibit a substantially higher Fe^3+^ coordination ability than TPPCDs (4.02 × 10^−4^ M) and DTPACDs (4.54 × 10^−4^ M), while the latter two show comparable Fe^3+^ coordination. This difference from the conjugated ligands’ structural properties: the dense O‐P‐O groups of HEDP are found to enhance coordination site density on CDs, offsetting free HEDP's lower log K (21.0) (vs. DTPA's 28.6) via a modulated microenvironment of immobilized ligands. Conversely, the bulky triphenyl moiety of TPP is shown to impose steric hindrance, limiting Fe^3+^ accessibility, whereas DTPA's high log K is counteracted by low surface loading density due to rigid chelation. These findings establish that ligand coordination chemistry (functional groups, steric effects) dually regulates CDs‐Fe nanozymes, demonstrating that free‐ligand log K alone cannot guide nanozyme design. The balance between coordination affinity and capacity is required to optimize catalytic performance.

X‐ray photoelectron spectroscopy (XPS) was further employed to analyze the surface composition and electronic states of HEDPCDs‐Fe. The survey spectrum of HEDPCDs‐Fe confirms the presence of C, N, O, P, and Fe, indicating successful iron incorporation (Figure S4). The high‐resolution Fe 2p spectrum (Figure 2d) was deconvoluted into two spin‐orbit doublets. The dominant peaks at 710.68 eV (Fe^3+^ 2p_1/2_) and 723.98 eV (Fe^3+^ 2p_3/2_) are accompanied by additional components at 708.93 and 722.78 eV, which are assigned to Fe‐P species, indicating chemical interaction between Fe and P in the HEDPCDs‐Fe. The C 1s and N 1s spectra (Figure S5) suggest that the HEDPCDs‐Fe retain the basic structural features of HEDP. Moreover, the P 2p spectrum (Figure 2e) shows a peak at 132.18 eV corresponding to P‐O bonds, along with two lower‐energy peaks at 128.18 and 127.18 eV, further confirming Fe‐P coordination. The O 1s spectrum further supports metal ions coordination by the HEDP ligand on the CDs (Figure 2f). Based on this analysis, a coordination chemistry approach was proposed to synthesize CDs‐Fe nanozyme utilizing different chelating ligands. As illustrated in Figure 2g, HEDPCDs‐Fe achieve metal coordination through their surface‐bound HEDP moieties, analogous to the HEDP‐Fe complex. Similarly, coordination in TPPCDs‐Fe and DTPACDs‐Fe is accomplished via their respective functionalized TPP and DTPA ligands.

### 2.4 POD‐Mimicking Catalytic Activity of CDs‐Fe with Tailor‐Made Ligands

**FIGURE 3:**
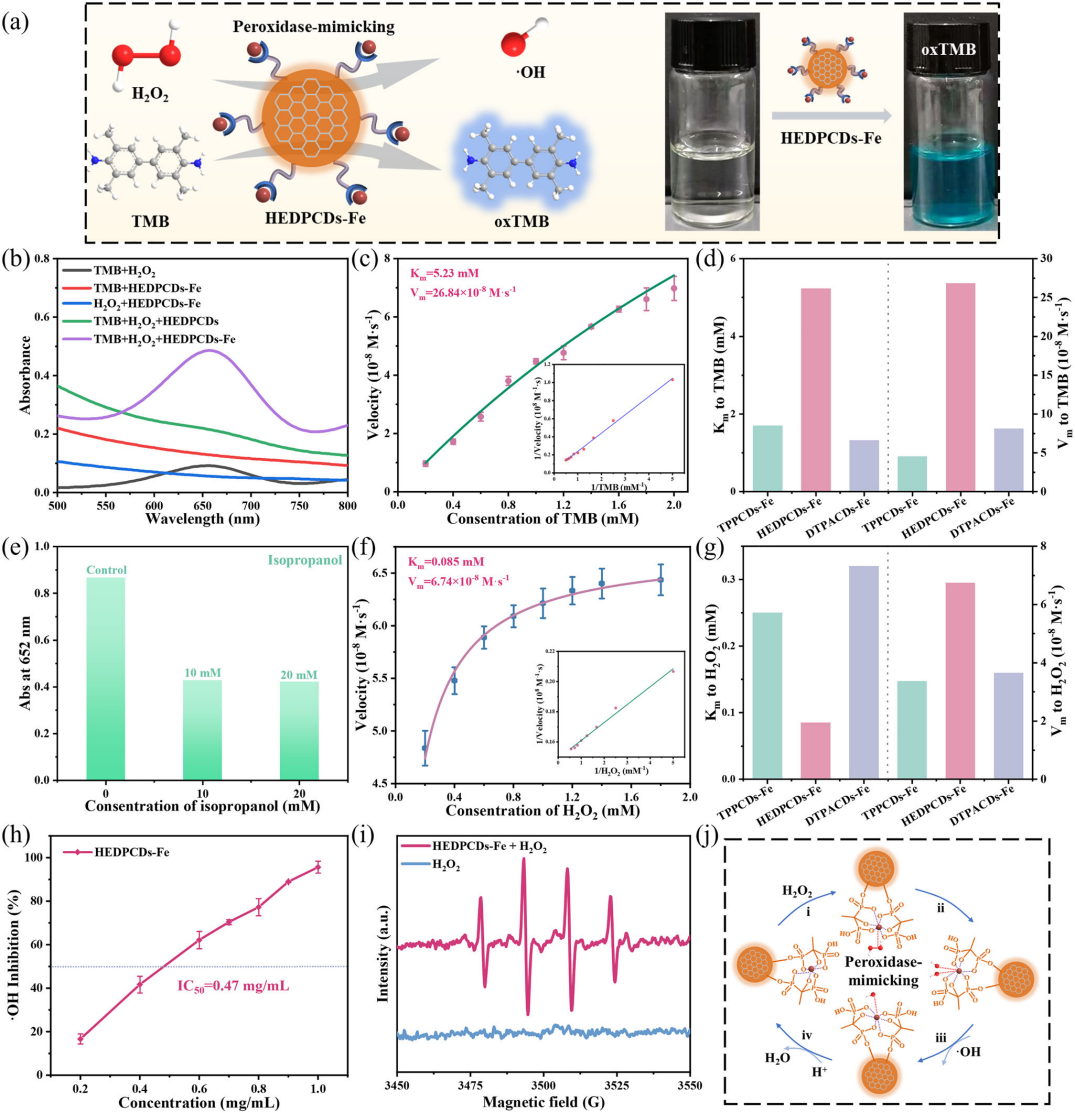
Ligands dominate POD‐mimicking activity. (a) POD‐mimicking of HEDPCDs‐Fe nanozymes by the oxidation of TMB. (b) UV–vis absorption spectra of TMB+H_2_O_2_, TMB+HEDPCDs‐Fe, H_2_O_2_+HEDPCDs‐Fe, TMB+H_2_O_2_+HEDPCDs, and TMB+H_2_O_2_+HEDPCDs‐Fe. Steady‐state kinetic analysis of HEDPCDs‐Fe for (c) TMB and (f) the H_2_O_2_ substrate. The K_m_ and V_m_ for TPPCDs‐Fe, HEDPCDs‐Fe, and DTPACDs‐Fe were compared using (d) TMB and (g) H_2_O_2_ as substrates. (e) The absorbance at 652 nm of TMB/H_2_O_2_/HEDPCDs‐Fe solution after adding different amounts of isopropanol. (h) ·OH inhibition rate of HEDPCDs‐Fe nanozymes at different concentrations. (i) The EPR spectra of H_2_O_2_ and HEDPCDs‐Fe + H_2_O_2_. (j) The proposed reaction mechanism of POD‐mimicking nanozymes. Data are presented as mean ± SD (*n* = 3 independent experiments).

After confirming that the CDs‐Fe structures share a central Fe^3+^ but are coordinated by distinct ligands, a systematic evaluation of their catalytic properties as nanozymes toward H_2_O_2_ was conducted. The POD‐mimicking activity was initially assessed using a TMB‐based colorimetric assay (Figure 3a). The UV–vis absorption spectra revealed that HEDPCDs‐Fe possesses higher activity compared to HEDPCDs alone, which showed minimal activity (Figure 3b). These results establish that Fe^3+^ incorporation is essential for catalytic function. Furthermore, the catalytic behavior of HEDPCDs‐Fe depends on pH and temperature, mirroring characteristics of natural enzymes (Figure S6a,b). Moreover, the POD‐mimicking activity was tested over 20 continuous catalytic cycles. The relative activity of HEDPCDs‐Fe retained 93% of the initial activity (Figure S6c), demonstrating excellent reusability. HEDPCDs‐Fe nanozymes stored at room temperature for 60 days retained ∼92% of the original catalytic activity (Figure S6d), indicating good long‐term stability.

Additionally, a quantitative assessment of the POD‐mimicking performance toward H_2_O_2_ was performed using steady‐state kinetics. When TMB was used as the substrate, HEDPCDs‐Fe exhibited a lower Michaelis constant (K_m_ = 5.23 mm) and a higher maximum velocity (V_m_ = 26.84 × 10^−8^ M s^−1^) than TPPCDs‐Fe and DTPACDs‐Fe (Figure 3c,d), indicating both a superior substrate affinity and a faster catalytic turnover. Similarly, with H_2_O_2_ as the substrate, HEDPCDs‐Fe showed a significantly lower K_m_ (0.085 mm) and a high V_m_ (6.74 × 10^−8^ M s^−1^) (Figure 3f,g). The minimal K_m_ value reflects exceptionally strong affinity for H_2_O_2_, facilitating efficient substrate capture, while the high V_m_ confirms rapid catalytic turnover. Overall, the superior reactivity and affinity of HEDPCDs‐Fe for both TMB and H_2_O_2_ establish it as the most effective POD‐mimicking nanozyme among those tested (Figure 3d,g; Figure S7). The HEDPCDs‐Fe nanozyme demonstrated superior substrate affinity for both TMB and H_2_O_2_ relative to other reported nanozymes (Table S1), confirming its high POD‐mimicking activity [23, 35, 36, 37]. The ligand‐regulation strategy of regulating chelating metal ions makes the coordination environment clear, which is different from other nanozymes.

To identify the reactive intermediates in the catalytic mechanism, isopropanol was used as a hydroxyl radical (·OH) scavenger. The effect of isopropanol on the POD‐mimicking activity was explored using HEDPCDs‐Fe. As depicted in Figure 3e, the characteristic absorption peak of oxTMB at 652 nm decreased progressively with increasing isopropanol concentration. These results identify the ·OH radical as the pivotal intermediate responsible for TMB oxidation. The marked suppression of oxTMB upon ·OH scavenging provided direct evidence that HEDPCDs‐Fe facilitates ·OH generation from H_2_O_2_, thereby enabling efficient TMB oxidation. As shown in Figure 3h, HEDPCDs‐Fe has significant ·OH scavenging activity with an IC_50_ value of 0.47 mg/mL. The result provides direct evidence for the antioxidant activity of HEDPCDs‐Fe, which is more in line with the intrinsic catalytic characteristics of the material compared with the DPPH scavenging assay. The electron paramagnetic resonance (EPR) spectra in Figure 3i provide direct evidence of ·OH generation by HEDPCDs‐Fe nanozymes. Based on these experimental results and existing literature, the potential catalytic reaction mechanism was proposed (Figure 3j) [56]. The H_2_O_2_ molecule first adsorbs onto the Fe ion site in the HEDPCDs‐Fe (i). At this single Fe site, the activated H_2_O_2_ undergoes homolytic dissociation into two hydroxyl groups (ii), followed by the desorption of one as a ·OH (iii) [57]. The remaining adsorbed hydroxyl group then reacts with protons in the acidic environment, leading to the adsorption of the H_2_O molecule (iv). Final desorption of H_2_O restores the metal active site to its initial catalytic state [58].

### 2.5 UV‐Induced OXD‐Mimicking Catalytic Activity of CDs‐Fe with Tailor‐Made Ligands

**FIGURE 4:**
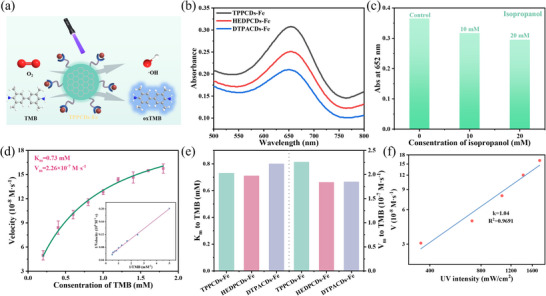
UV‐induced OXD‐mimicking activity of TPPCDs‐Fe. (a) OXD‐mimicking of TPPCDs‐Fe nanozymes by the oxidation of TMB. (b) Comparison of OXD‐mimicking relative activities of different CDs‐Fe nanozymes. (c) The absorbance at 652 nm of TMB/H_2_O_2_/TPPCDs‐Fe solution after adding different amounts of isopropanol. (d) Steady‐state kinetic analysis of TPPCDs‐Fe for TMB substrate. (e) The K_m_ and V_m_ for different CDs‐Fe nanozymes were compared using TMB as substrates. (f) The log‐log fitting of the initial reaction rate vs. UV intensity. Data are presented as mean ± SD (*n* = 3 independent experiments).

The UV‐induced OXD‐mimicking behavior of the CDs‐Fe nanozymes was verified by the TMB colorimetric reaction. As illustrated in Figure 4a, TPPCDs‐Fe acts as the catalytic center, which, under 365 nm UV excitation, activates O_2_ to generate ·OH that oxidizes TMB to the colored oxTMB. This transformation demonstrates the conversion of photonic energy into chemical energy, thereby driving the catalytic reaction. The UV‐induced oxidation activities of TPP‐CDs‐Fe, HEDP‐CDs‐Fe, and DTPA‐CDs‐Fe were compared by monitoring TMB oxidation (Figure 4b). The results show that TPPCDs‐Fe generates a significantly more intense oxTMB absorption peak at 652 nm than HEDPCDs‐Fe and DTPACDs‐Fe, indicating its superior UV‐induced OXD‐mimicking activity. These activity differences are attributed to variations in both the Fe‐ligand coordination environments and the conjugated electronic characteristics of the CDs, which collectively influence oxygen activation and electron transfer kinetics. The superior performance of TPPCDs‐Fe originates from the abundant π‐electrons provided to CDs by the conjugated aromatic structure of TPP, which facilitates electron transition and transfer under UV excitation, thereby enhancing activation. Interestingly, TPPCDs‐Fe exhibited a comparatively high affinity for TMB substrate, indicating robust OXD‐mimicking activity relative to other nanozymes (Table S2) [35, 36, 37, 59]. Meanwhile, the optimal reaction pH of TPPCDs‐Fe was determined at 3.0 (Figure S8a). After UV irradiation for 5 min, the TPPCDs‐Fe nanozymes maintained over 80% of their initial catalytic activity, which indicates good UV stability (Figure S8b). Also, TPPCDs‐Fe nanozymes stored at room temperature for 60 days retained ∼91% of their original catalytic activity (Figure S8c), indicating good long‐term stability. These new results strongly support the potential practical application of CDs‐Fe nanozymes.

To elucidate the critical reactive species in the catalytic process, isopropanol was added as a ·OH radical scavenger. Figure 4c shows that the oxTMB absorption peak at 652 nm gradually decreased as the concentration of isopropanol increased from 0 to 20 mm, confirming ·OH is the core intermediate in the UV‐induced oxidation of TMB. The suppression of TMB oxidation by isopropanol corroborates that ·OH generated through nanozyme UV activation is responsible for the efficient TMB oxidation.

To preclude contributions from substrate autoxidation or photobleaching, a control experiments (encompassing dark reactions and substrate‐only irradiation) were conducted. Markedly diminished product yields were noted in the dark control (Figure S8d), demonstrating that the reaction is oxygen‐dependent and the formation of the main product is UV‐induced. Steady‐state kinetic studies were further performed to quantitatively assess the UV‐induced OXD‐mimicking activity of TPPCDs‐Fe toward O_2_. The measured K_m_ and V_m_ values for TPPCDs‐Fe with TMB were 0.73 mm and 2.26 × 10^−7^ M s^−1^, respectively, outperforming HEDPCDs‐Fe and DTPACDs‐Fe nanozymes (Figure 4d,e; Figure S9). Log‐log fitting of the initial reaction rate vs. UV intensity yielded a slope of 1.04 (R^2^ = 0.9691 for TPPCDs‐Fe, Figure 4f), indicating a single‐photon‐dominated process. These kinetic parameters indicate a stronger affinity for TMB and a higher catalytic efficiency of TPPCDs‐Fe. The outstanding UV‐induced OXD‐mimicking activity of TPPCDs‐Fe originates from the conjugated aromatic structure of TPP, which enriches the CDs with π‐electrons. This electronic feature promotes UV‐induced electron excitation and transfer, thereby accelerating more oxygen activation.

### 2.6 Synergistic Enhancement of Enzyme‐Mimicking Activities in CDs‐Fe Nanozymes via UV‐Enhanced POD‐OXD

**FIGURE 5:**
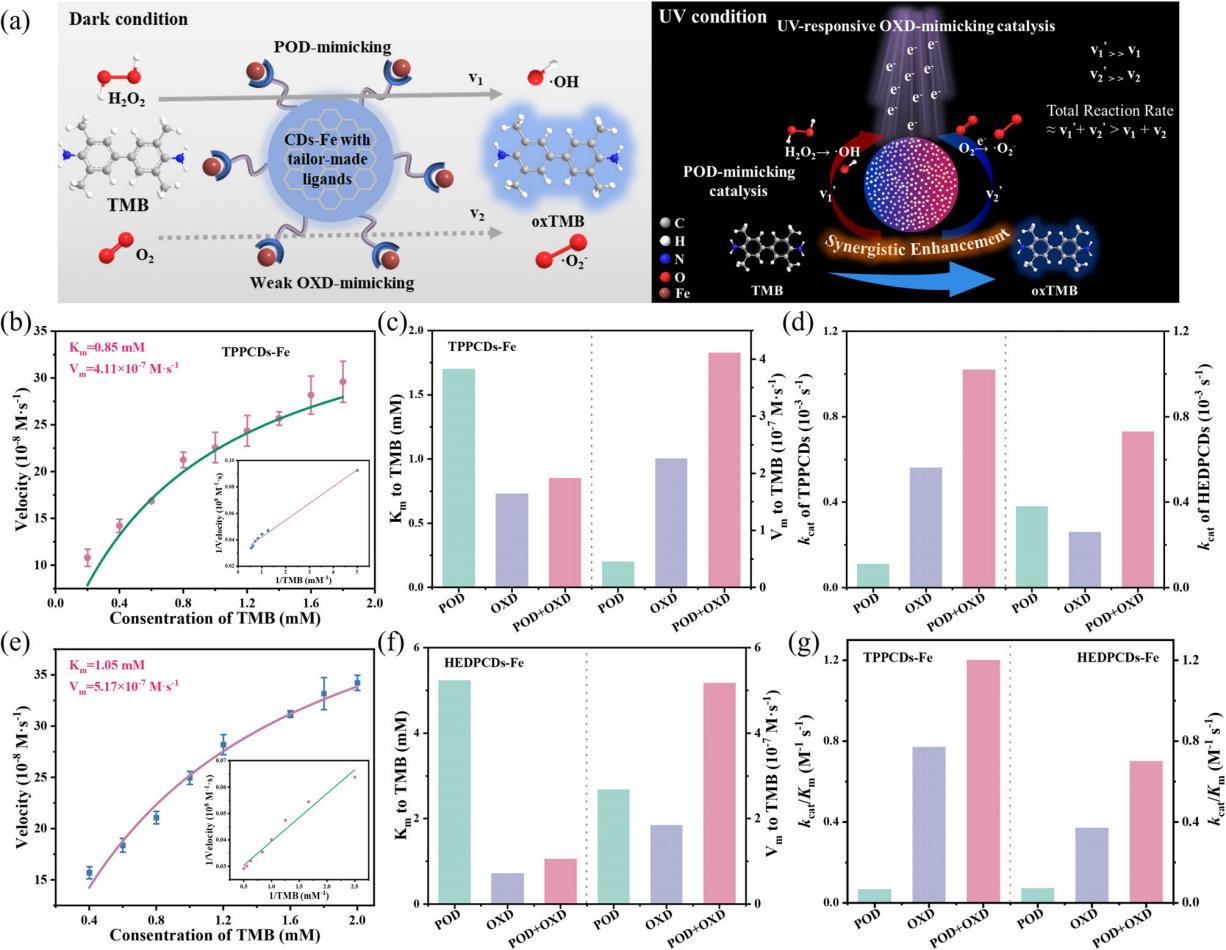
Synergistic enhancement of catalytic activities in CDs‐Fe nanozymes via UV‐enhanced POD‐OXD‐mimicking activity. (a) Schematic illustration of UV‐enhanced POD‐OXD synergistic enhancement of HEDPCDs‐Fe. Steady‐state kinetic analysis of (b) TPPCDs‐Fe and (e) HEDPCDs‐Fe for TMB substrate. The K_m_ and V_m_ for TPPCDs‐Fe (c), HEDPCDs‐Fe (f) were compared using TMB substrate. The (d) *k*_cat_ and (g) *k*_cat_/*K*_m_ for TPPCDs‐Fe and HEDPCDs‐Fe to POD‐, OXD‐, POD‐OXD‐mimicking activity. Data are presented as mean ± SD (*n* = 3 independent experiments).

After confirming that the CDs‐Fe nanozymes possess both POD‐mimicking activity and UV‐induced OXD‐mimicking activity, their UV‐enhanced POD‐OXD synergistic catalytic performance was systematically evaluated. The synergistic enhancement of POD‐mimicking and UV‐induced OXD‐mimicking activities in HEDPCDs‐Fe nanozymes can be attributed to a unique UV‐driven electron transfer mechanism (Figure 5a). Under dark conditions, the HEDPCDs‐Fe nanozyme mediates POD‐mimicking catalysis (yielding ·OH at rate v_1_) but shows only weak OXD‐mimicking activity, leading to a low corresponding reaction rate v_2_ for generating ·O_2_^−^ and oxTMB. Upon UV irradiation, the HEDPCDs‐Fe act as a photosensitizer, generating electron–hole pairs. The photogenerated electrons are efficiently injected into the Fe catalytic centers via the interfacial coordination bonds, thereby markedly accelerating the Fe^3+^/Fe^2+^ redox cycle. This process not only boosts the POD‐mimicking activity (v_1_^'^>v_1_) for enhanced ·OH generation from H_2_O_2_ but also concurrently activates the OXD‐mimicking pathway by facilitating the reduction of ambient O_2_ to ·O_2_^−^ (v_2_^'^>v_2_). The collaborative action of these simultaneously produced ROS from dual pathways results in a synergistic catalytic output, substantially exceeding the sum of their individual contributions (v_1_^'^+v_2_^'^>v_1_+v_2_).

Kinetic revealed that TPPCDs‐Fe exhibited K_m_ and V_m_ values of 0.85 mm and 4.11 × 10^−7^ M s^−1^(Figure 5b), respectively; HEDPCDs‐Fe showed values of 1.05 mm and 5.17 × 10^−7^ M s^−1^(Figure 5e). To further investigate the synergistic effect, the kinetic behavior of TPPCDs‐Fe was compared across three catalytic modes (POD‐mimicking, UV‐induced OXD‐mimicking, and UV‐enhanced POD‐OXD synergistic). As illustrated in Figure 5c, the K_m_ value in the synergistic mode was significantly lower than in either single mode, while the V_m_ value was markedly higher. This indicates that the synergistic effect enhances both substrate affinity and catalytic rate. A similar trend was observed for HEDPCDs‐Fe in Figure 5f, further confirming the universality of this synergistic mechanism. The variation in enhancement magnitude among different nanozymes is attributed to differences in their microstructures, which modulate the synergistic efficacy. The markedly enhanced catalytic constant (*k*_cat_) and catalytic efficiency (*k*_cat_/*K*_m_) in the dual POD + OXD system reflect a strong synergistic effect (Figure 5d, g; Table S3). TPPCDs‐Fe favor OXD, while HEDPCDs‐Fe show balanced activities. These differences arise from ligand modulation, demonstrating that ligand engineering enables rational optimization of multifunctional nanozyme performance. The proposed mechanism is strongly supported by experimental evidence. The EPR spectra confirmed the formation of ·OH by the HEDPCDs‐Fe nanozymes. As illustrated in Figure S10, a distinct DMPO‐OH quartet signal was observed in the presence of H_2_O_2_. This signal was further enhanced under 365 nm UV irradiation, providing direct evidence of the synergistic catalytic performance from the POD‐mimicking pathway and the UV‐induced OXD‐mimicking pathway.

Above all, the synergistic interaction of UV‐enhanced POD‐OXD‐mimicking activities significantly enhances the oxidative capability of CDs‐Fe nanozymes toward TMB, as evidenced by improved substrate affinity (reduced K_m_) and accelerated catalytic rates (increased V_m_). Although the degree of synergistic enhancement varies with the specific CDs‐Fe nanozymes due to structural differences, the universal dual‐path catalytic coupling mechanism was verified across all systems, providing a valuable theoretical foundation for designing highly efficient UV‐induced nanozymes.

### 2.7 Relationship of Ligand Structure and Catalytic Performance of CDs‐Fe Nanozyme

The catalytic performance of CDs‐Fe nanozymes is tightly regulated by the chemical nature of surface ligands, as reflected by key kinetic parameters (K_m_, V_m_, *k*_cat_, *k*_cat_/K_m_) for POD‐mimicking, UV‐induced OXD‐mimicking, and synergistic POD+OXD activities (Tables S1–S3). For POD‐mimicking activity, ligand coordination atoms, steric hindrance, and electronic effects collectively govern kinetic behavior. Regarding substrate affinity (K_m_), HEDPCDs‐Fe exhibits the lowest K_m_ for H_2_O_2_ (0.085 mm, Table S1), attributed to the strong electron donation of HEDP's O‐donor phosphonate groups, and then enhancing electrostatic attraction between Fe^3+^ active sites and polar H_2_O_2_. In contrast, TPPCDs‐Fe (K_m_ = 0.25 mm) and DTPACDs‐Fe (K_m_ = 0.32 mm) show weaker H_2_O_2_ binding, due to TPP's bulky aromatic steric hindrance and DTPA's rigid hexadentate chelate restricting substrate access. For the hydrophobic TMB substrate, TPPCDs‐Fe (K_m_ = 1.17 mm) and DTPACDs‐Fe (K_m_ = 1.32 mm) benefit from *π–π* stacking (TPP's conjugated rings) or weak hydrophobic interactions (DTPA's amides), while HEDP's lack of hydrophobic moieties results in poor TMB affinity (K_m_ = 5.23 mm). In terms of maximum reaction rate (V_m_), HEDPCDs‐Fe achieves the highest value (26.84 × 10^−8^ Μ s^−1^, Table S1), as O‐donor coordination lowers the Fe^3+^/Fe^2+^ redox potential (facilitating H_2_O_2_ cleavage) and its compact structure exposes more active sites. TPPCDs‐Fe (4.54 × 10^−8^ Μ s^−1^) is limited by steric hindrance, while DTPACDs‐Fe (8.10 × 10^−8^ Μ s^−1^) balances stability and activity via rigid N/O chelation. Catalytic efficiency (*k*_cat_/K_m_) further highlights ligand‐specific advantages: HEDPCDs‐Fe dominates for H_2_O_2_ (1.12 Μ^−1^ s^−1^, Table S3) due to ultralow K_m_, while DTPACDs‐Fe leads for TMB (0.14 Μ^−1^ s^−1^) via balanced affinity and turnover.

In UV‐induced OXD‐mimicking activity (TMB as substrate), kinetic regulation differs markedly. All nanozymes exhibit similar Km values (0.71–0.80 mm, Table S2), as UV‐activated Fe sites promote TMB adsorption via photoinduced electrostatic interactions, overriding ligand‐dependent hydrophobic effects. However, V_m_ and *k*_cat_/K_m_ are strongly dependent on ligand conjugation: TPPCDs‐Fe achieves the highest V_m_ (22.60 × 10^−8^ Μ s^−1^) and catalytic efficiency (0.77 M^−1^ s^−1^, Table S3), as TPP's conjugated aromatic rings act as electron reservoirs, reducing electron–hole recombination and accelerating electron transfer to O_2_. HEDPCDs‐Fe and DTPACDs‐Fe, lacking conjugated moieties, show comparable but lower activity (V_m_≈18.4–18.5 × 10^−^^8^ Μ s^−^^1^, *k*_cat_/K_m_≈0.37–0.51 M^−1^ s^−1^).

Notably, TPPCDs‐Fe exhibits the strongest synergistic POD+OXD‐mimicking activity, with *k*_cat_ (1.02 × 10^−3^ s^−1^) and *k*_cat_/K_m_ (1 M^−1^ s^−1^, Table S3) significantly exceeding single‐pathway activities. This synergy stems from TPP's dual structural merits: N/P coordination sustains Fe^3+^‐mediated POD activity, while conjugated rings boost UV‐driven OXD activity. In contrast, HEDPCDs‐Fe (O‐donor, no conjugation) and DTPACDs‐Fe (rigid chelate, no conjugation) cannot optimize both pathways simultaneously, resulting in weaker synergism (*k*_cat_/K_m_≈0.70 M^−1^ s^−1^).

Collectively, these results demonstrate that ligand structure tailors nanozyme kinetic behavior (Table S4). HEDP (compact O‐donor) optimizes POD activity via rate‐driven enhancement, DTPA (rigid N/O chelate) favors POD activity via affinity balance, and TPP (conjugated N/P ligand) dominates OXD and synergistic catalysis. This rational ligand design strategy provides a versatile approach to customize nanozymes for specific catalytic demands.

## 3 Conclusions

In summary, this study successfully synthesized a series of CDs–Fe nanozymes functionalized with tailor‐made ligands (TPP, HEDP, and DTPA) via a combination of hydrothermal carbonization and coordination chemistry. The main findings demonstrate that the ligand moieties are a decisive factor in tuning the catalytic performance. HEDPCDs‐Fe exhibited the highest POD‐mimicking activity, whereas TPPCDs‐Fe showed superior UV‐induced OXD‐mimicking capability. Remarkably, a pronounced UV‐enhanced POD‐OXD synergistic effect was observed between these activities: UV‐generated ROS accelerated H_2_O_2_ decomposition in POD‐mimicking reactions, and conversely, POD‐mediated H_2_O_2_ activation promoted more efficient UV‐induced O_2_ reduction. This synergy was quantitatively confirmed by enhanced kinetic parameters, specifically a lower K_m_ (1.05 mm) and higher V_m_ (5.17 × 10^−7^ M s^−1^) in the synergistic catalytic mode. This work establishes a ligand‐regulation strategy for designing high‐performance nanozymes, providing a new pathway for synergistic catalytic material design and synthesis.

## Conflicts of Interest

The authors declare no conflicts of interest.

## Supporting information




**Supporting File**: advs74023‐sup‐0001‐SuppMat.docx.

## Data Availability

The data that support the findings of this study are available in the supplementary material of this article.
