# Supplementary material for: Synergistic Enhancement of Catalytic Activities in Ligand‐Regulated Carbon Dots‐Ferric Ion Nanozymes via UV‐Enhanced Peroxidase‐Oxidase
Source: Adv Sci (Weinh). 2026 Feb 28;13(20):e19424. doi: 10.1002/advs.202519424 (PMC13067779; doi:10.1002/advs.202519424)
Supplement: Supplementary file 1 — Supporting File: advs74023‐sup‐0001‐SuppMat.docx. [file ADVS-13-e19424-s001.docx]

**Supporting Information**

**Synergistic Enhancement of Catalytic Activities in Ligand-Regulated Carbon Dots-Ferric Ion Nanozymes via UV-Enhanced Peroxidase-Oxidase**

*Xiangli Li, Jiaxin Dong, Dechang Jia, Yu Zhou, and Baoqiang Li**

**Experimental Section**

*Chemicals and Materials*: All chemicals were used as received without further purification. Acrylamide (AM) was purchased from Aladdin (Shanghai, China). Allyltriphenylphosphonium bromide (TPP), diethylenetriaminepentaacetic acid (DTPA), 1-hydroxyethane-1,1-diphosphonic acid (HEDP), and deuterium oxide (D_2_O) were obtained from Energy Chemical (Anhui, China). Ammonium persulfate (APS) was sourced from Fuchen Chemical Reagent (Tianjin, China). Sodium acetate anhydrous and ethylenediaminetetraacetic acid (EDTA) came from Tianjin Chemical Reagent Co., Ltd. (Tianjin, China). Tetramethylbenzidine (TMB) was bought from Macklin (Shanghai, China). Acetic acid was purchased from Tianjin FUYU Fine Chemical Co., Ltd. (Tianjin, China). Iron trichloride (FeCl_3_) was purchased from Sinopharm Chemical Reagent Co., Ltd. (Shanghai, China). And Hydrogen peroxide (H_2_O_2_, 30%) was purchased from Yongchang Reagent (Heilongjiang, China). All solutions were prepared with deionized water (18.2 MΩ·cm resistivity) from a Milli-Q water purification system. Dialysis bags (MWCO = 500 Da) were purchased from Solarbio (USA).

*Synthesis of CDs-Fe* *with tailor-made ligands based on coordination chemistry*:

Firstly, the synthesis of TPPCDs was adapted from our previous literature^[1]^ with modifications to the precursors: 1 mmol TPP, 28 mmol AM, and 0.06 mmol APS. Using an analogous procedure, HEDPCDs and DTPACDs were prepared by substituting TPP with HEDP and DTPA, respectively. Subsequently, purified TPPCDs solution (2 mg/mL) was incubated overnight with 1 mM FeCl₃. To remove free iron ions, the resulting mixture was dialyzed (MWCO = 500 Da) for 24 hours. Finally, TPPCDs-Fe (TPPCDs-Fe³⁺) powder was obtained by lyophilization. HEDPCDs-Fe and DTPACDs-Fe were prepared identically; these materials, distinguished by their custom ligands, are collectively designated as CDs-Fe with tailor-made ligands.

*Qualitative characterization of functional groups and coordination bonds of CDs-Fe with tailor-made ligands*: The morphologies of CDs with tailor-made ligands and CDs-Fe nanozyme were observed by a Talos F200X TEM (ThermoFisher). Surface functional groups of TPPCDs, HEDPCDs, and DTPACDs were identified by Fourier-transform infrared (FTIR) spectroscopy (Thermo Electron Corporation, Nicolet 380). Furthermore, X-ray photoelectron spectroscopy (XPS, ESCALAB 250, Al Kα) was employed to confirm the surface properties. The metal loadings of CDs-Fe with tailor-made ligands were measured by ICP-MS (Thermo Scientific iCAP7400). The electron paramagnetic resonance (EPR) spectroscopy was conducted on an EPR200-Plus spectrometer.

*Characterization*: The UV-vis spectra and fluorescence spectra were measured by FL970 spectrofluorometer (Techcomp, China) and TU 1901 spectrophotometer (Beijing General Analytical Instrument, China), respectively. The UV-vis absorbance spectra were measured to evaluate the UV-induced POD-mimicking and OXD-mimicking activities, and fluorescence spectra were measured to evaluate the optical properties of TPPCDs, HEDPCDs, and DTPACDs.

*Quantitative characterization of affinity ability of CDs-Fe with tailor-made ligands*: The affinity of TPPCDs, HEDPCDs, and DTPACDs for Fe^3+^ was assessed via fluorescence quenching assays. Different concentrations of Fe^3+^ (0-200 μM) were added to the CDs with tailor-made ligand solutions to measure the change of fluorescence intensity of TPPCDs, HEDPCDs, and DTPACDs. The K of CDs-Fe with tailor-made ligand complexes was calculated by the Benesi-Hildebrand equation (1)^[2]^:

$\frac{1}{F-F_{0}}=\frac{a}{a-b}\left[ \frac{1}{K\left[ M \right]}+1 \right]$ (1)

In this equation, $F$ and $F_{0}$ represent the fluorescence intensity of TPPCDs, HEDPCDs, and DTPACDs in the presence and absence of Fe^3+^, respectively. *[M]* is the concentration of Fe^3+^, and a and b are both constants. The K of TPPCDs, HEDPCDs, and DTPACDs were used to characterize the affinity between different CDs with tailor-made ligands and Fe^3+^.

***Enzyme-mimicking performances of CDs-Fe with tailor-made ligands***

*POD-mimicking catalytic activity assays*: POD-mimicking catalytic activity was assessed using a TMB oxidation assay. In detail, the reaction mixture was prepared in a 3.0 mL tube by combining 2.7 mL of NaOAc/HOAc buffer (pH 3.0) with 120 μL of TMB (20 mM), 150 μL of H_2_O_2_ (50 mM), and 30 μL of the respective CDs-Fe solution (TPPCDs-Fe, HEDPCDs-Fe, or DTPACDs-Fe at 10 mg/mL). This yielded final concentrations of 0.8 mM TMB, 2.5 mM H_2_O_2_, and 100 μg mL^−1^ of the catalyst. After incubation at 37 °C for 5 min, the POD-mimicking catalytic activity was evaluated by monitoring the absorbance of oxTMB at 652 nm. All OXD activity assays were performed in an air-saturated buffer unless specified otherwise.

*Steady-state enzyme kinetic studies*: Before the kinetic analysis, the optimal pH for the TMB oxidation reaction catalyzed by the CDs-Fe was explored. Subsequently, the kinetic parameters, including the Michaelis constant (K_m_), maximum velocity (V_m_), and turnover number (TON), were calculated from the Michaelis-Menten saturation curve.

*Evaluation of the POD-mimicking catalytic activity of CDs-Fe nanozymes*: Kinetic assays were performed in NaOAc/HOAc buffer (0.1 M, pH 3.0) under two sets of conditions to determine the kinetic parameters for each substrate. In the first set, the reaction mixture contained a fixed concentration of catalyst (100 μg mL⁻¹ CDs-Fe) and H_2_O_2_ (2.5 mM) with varying TMB concentrations (0.2 to 2.0 mM). Conversely, the second set maintained a fixed concentration of the catalyst (100 μg mL⁻¹ CDs-Fe) and TMB (0.8 mM), while the concentration of H_2_O_2_ was varied (0.2 to 2.0 mM). All assays were performed three times. The K_m_ and V_m_ were calculated according to equation (2):

$\frac{\text{1}}{\text{V}_{\text{0}}}\text{=}\frac{\text{K}_{\text{m}}}{\text{V}_{\text{m}}\left[ \text{S} \right]}\text{+}\frac{\text{1}}{\text{V}_{\text{m}}}$ (2)

$\text{k}_{\text{cat}}\text{=}\text{V}_{\text{m}}\text{/(E)}$ (3)

Furthermore, the catalytic constant $\text{k}_{\text{cat}}$ was calculated using equation (3), where (E) represents the molar concentration of the metal within the nanomaterial.

*Evaluation of UV-induced* *OXD-mimicking enzyme activity of CDs-Fe nanozymes*: The OXD-mimicking activity of the CDs-Fe was assessed by monitoring the oxidation of the chromogenic substrate TMB. A standard assay mixture was prepared by combining 30 μL of CDs-Fe (10 mg/mL) and varying TMB concentrations (0.2 to 2.0 mM) with NaOAc/HOAc buffer (0.1 M, pH 3.0). Following 1 minute of irradiation under UV irradiation (365 nm, 10 W), the absorbance at 652 nm was measured to quantify the reaction product.

*Evaluating the UV-enhanced POD-OXD synergistic effect of CDs-Fe Nanozymes*: The kinetic parameters of UV-enhanced POD-OXD-mimicking activities of CDs-Fe nanozymes were also determined via TMB oxidation in NaOAc/HOAc buffer (0.1 M, pH 3.0). In the assay, the concentrations of the catalyst (100 μg mL⁻¹) and H_2_O_2_ (2.5 mM) were held constant, while the TMB concentration was varied from 0.2 to 2.0 mM. The absorbance of oxTMB at 652 nm was recorded after the reaction mixture was subjected to UV irradiation (365 nm, 10 W) for one minute.

*Evaluation of ·OH Scavenging ·OH Ability of CDs-Fe Nanozymes*: The ·OH scavenging ability of CDs-Fe nanozymes was assessed. A mixture was prepared containing FeSO_4_ (2 mL, 9.0 mM), ethanol-salicylic acid (1 mL, 9.0 mM), H_2_O_2_ (0.1 mL, 8.8 mM), and sample (1 mL). This solution was thoroughly blended and incubated at 37°C for 20 minutes, and then the absorbance at 510 nm was measured.

$$\text{·OH scavenging rate (\%)=}\frac{\text{A}_{\text{0}}\text{-}\left( \text{A}_{\text{x}}\text{-}\text{A}_{\text{x0}} \right)}{\text{A}_{\text{0}}}\text{×100}$$

where $\text{A}_{\text{0}}$ represents the absorbance of the blank control, $\text{A}_{\text{x}}$ is the absorbance of the sample, and $\text{A}_{\text{x0}}$ is the absorbance measured in the absence of H_2_O_2_. The half-maximal inhibitory concentration (IC_50_) for ·OH scavenging was determined via linear regression of a dose-response curve, where the scavenging rates were plotted against sample concentration.

*Statistical Analysis*: All experiments were performed in triplicate, except where specifically noted. The graphical representations include error bars corresponding to standard deviations. Results data are expressed as mean ± standard deviation (SD) derived from three independent replicates. Statistical analysis was conducted using OriginPro software (version 2021, OriginLab Inc., USA). The number of samples for each analysis was provided in each figure legend.


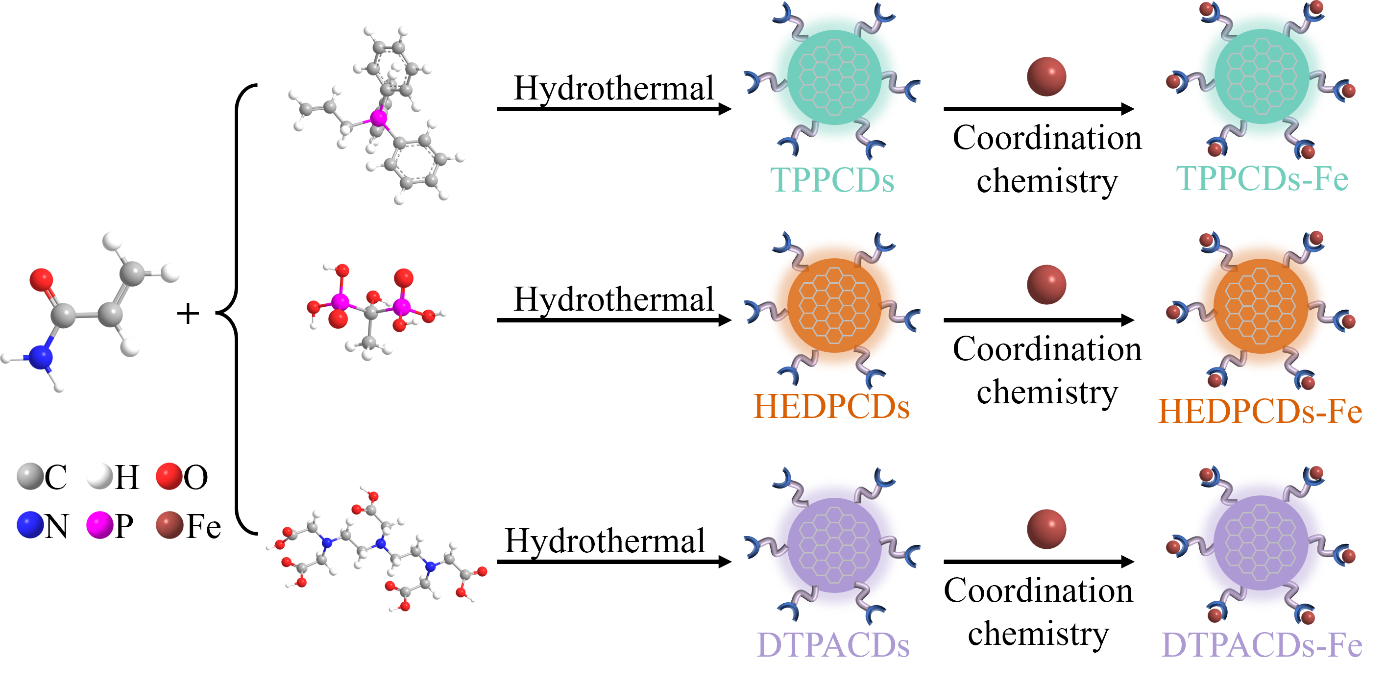


**Figure S1.** Schematic diagram of the synthesis of TPPCDs, HEDPCDs, and DTPACDs based on a coordination chemistry strategy.


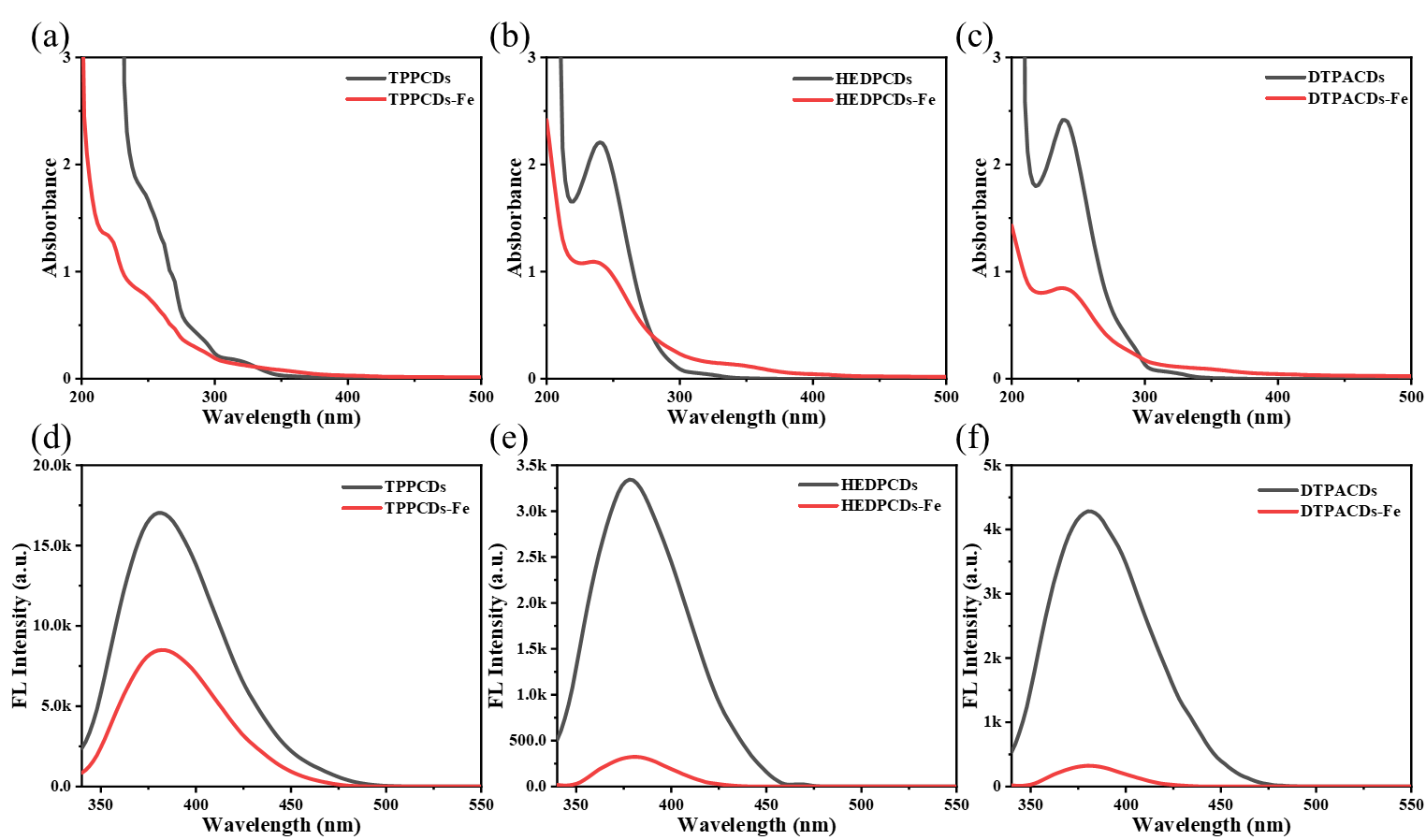


**Figure S2.** UV-vis absorption spectra of (a) TPPCDs, TPPCDs-Fe, (b)HEDPCDs, HEDPCDs-Fe, and (c) DTPACDs, DTPACDs-Fe. Fluorescence spectra of (d) TPPCDs, TPPCDs-Fe, (e)HEDPCDs, HEDPCDs-Fe, and (f) DTPACDs, DTPACDs-Fe.

**
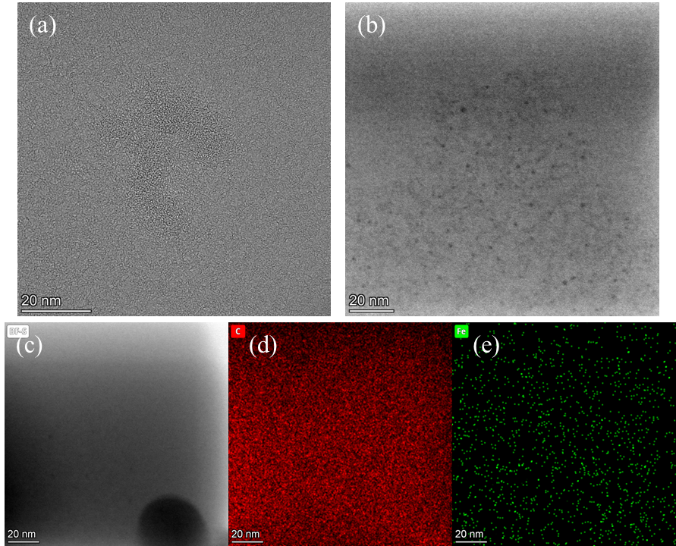
**

**Figure S3.** TEM images of (a) HEDPCDs and (b) HEDPCDs-Fe. The corresponding EDS elemental distribution of the C and Fe atoms in HEDPCDs-Fe nanozymes (c-e).

**Figure S4.** The XPS survey spectrum of HEDPCDs-Fe.


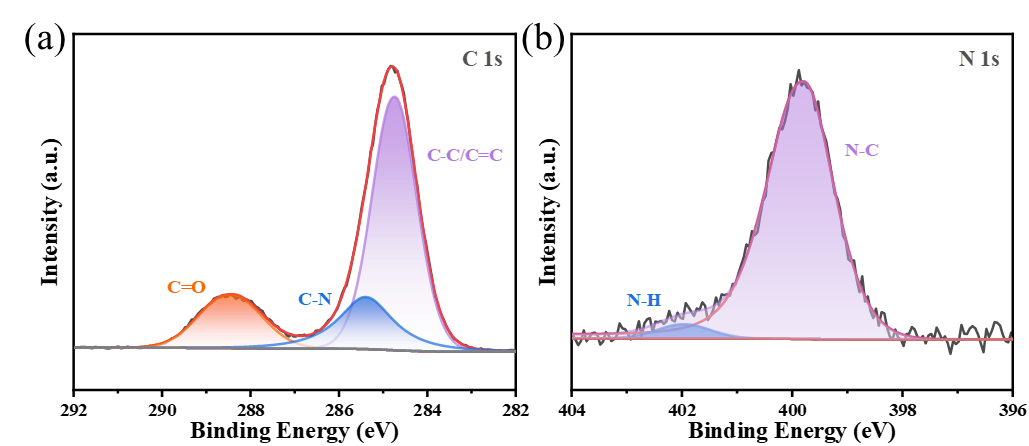


**Figure S5.** The (a) C 1s and (b) N 1s spectra of HEDPCDs-Fe.


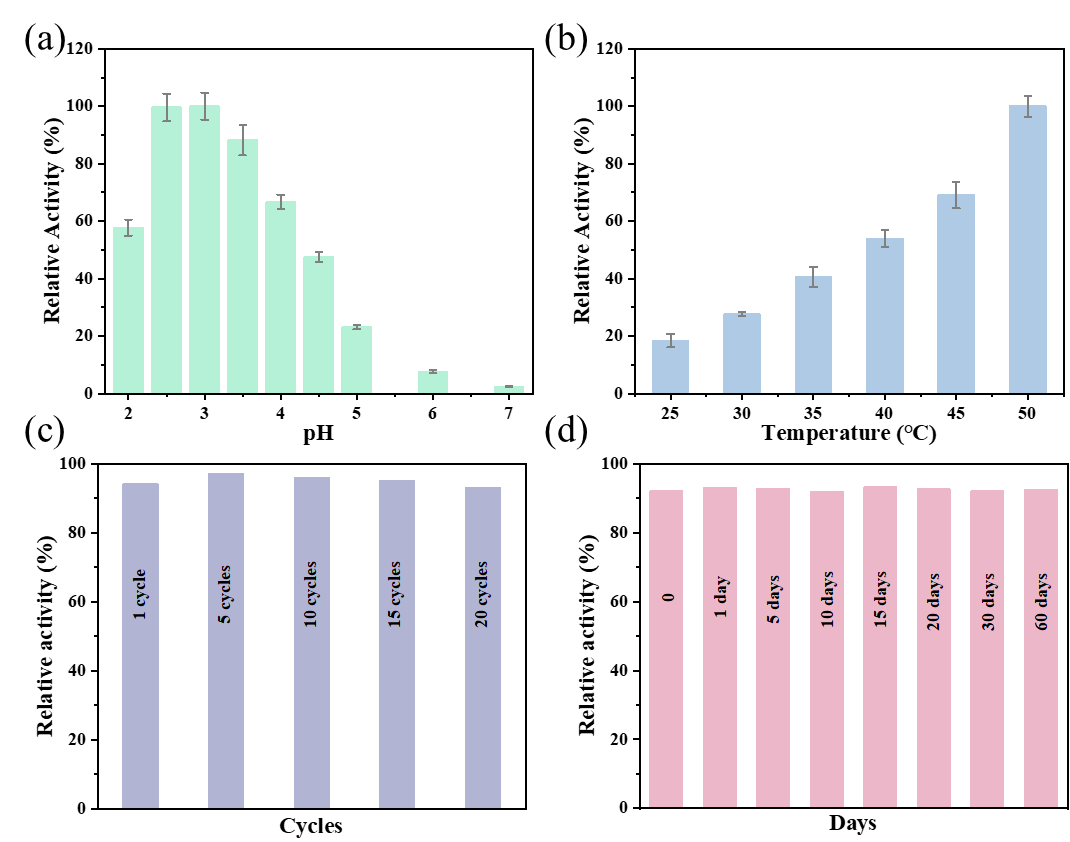


**Figure S6.** Relative POD-mimicking catalytic activity of HEDPCDs-Fe dependent on different conditions. (a) pH, (b) temperature, (c) Number of cycles, (d) Storage days. Data are presented as mean ± SD (n =3 independent experiments).


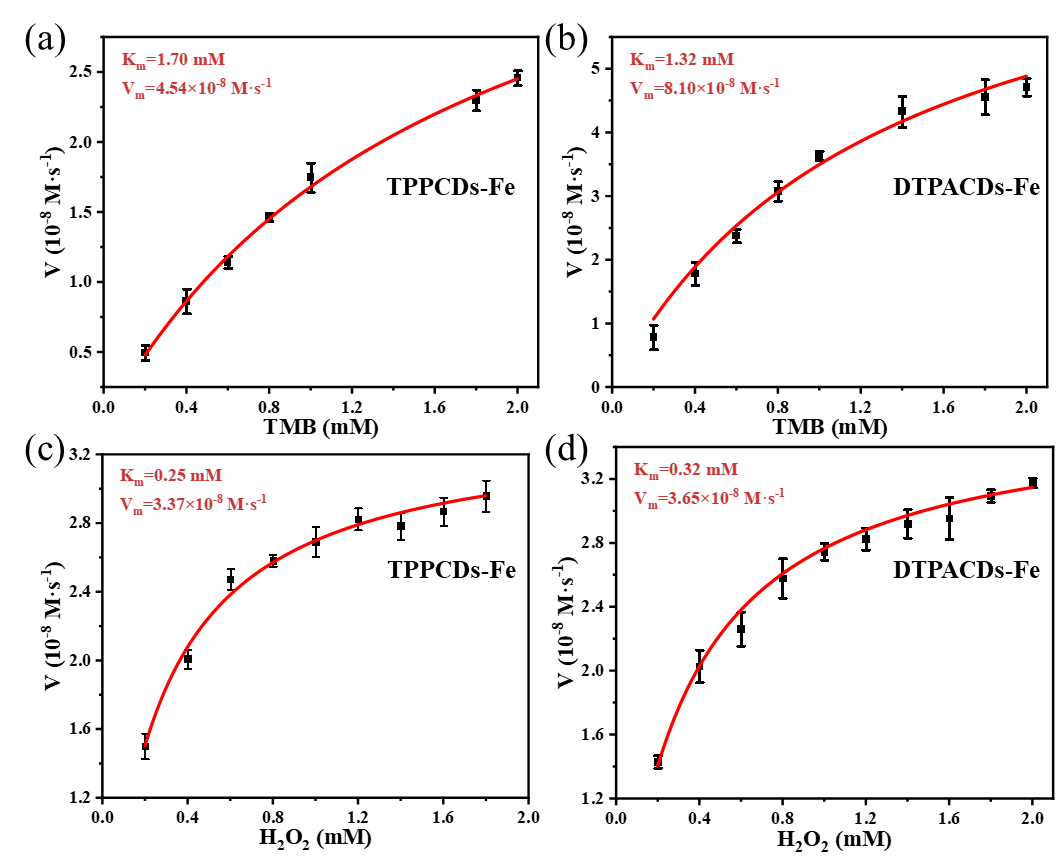


**Figure S7.** POD-mimicking steady-state kinetic analysis for TMB substrate of (a) TPPCDs-Fe and (b) HEDPCDs-Fe. Steady-state kinetic analysis for H_2_O_2_ substrate of (c) TPPCDs-Fe and (d) HEDPCDs-Fe. Data are presented as mean ± SD (n =3 independent experiments).


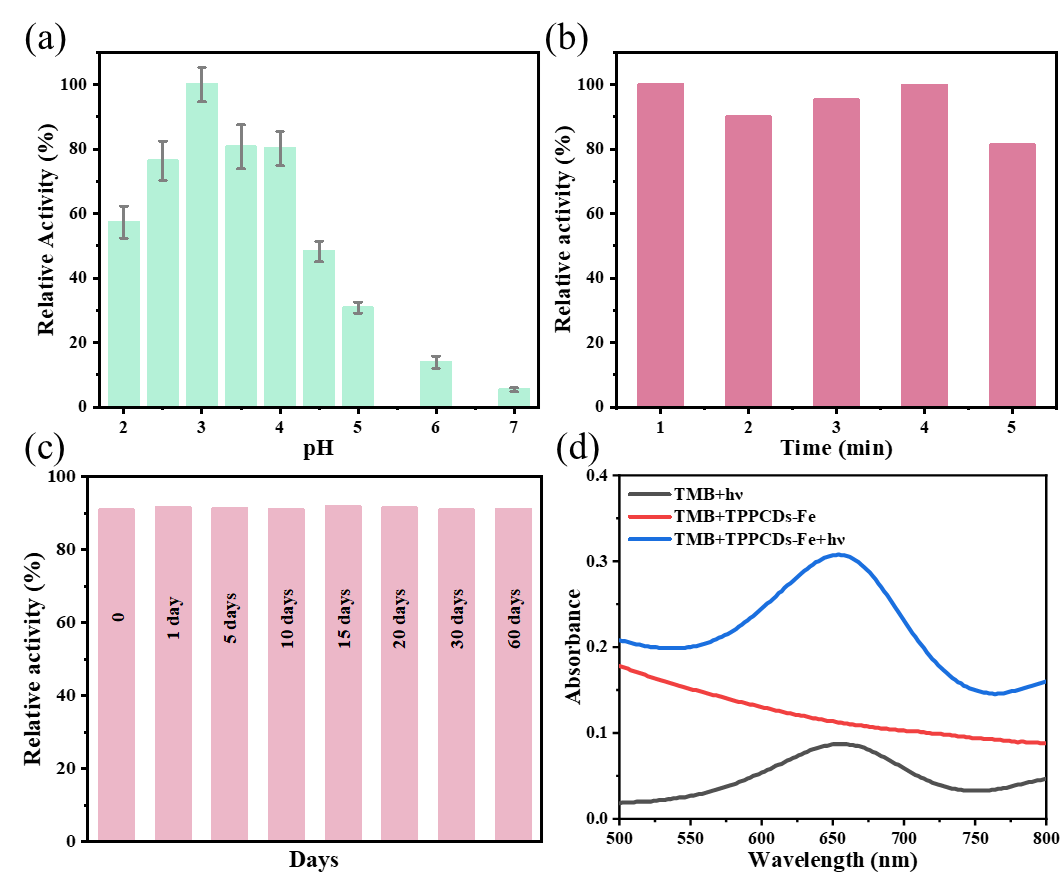


**Figure S8.** Effect of different reaction conditions on OXD-like relative activity of TPPCDs-Fe nanozymes. (a) pH, (b) UV irradiation time, (c) Storage days, (d) Comparison of OXD-mimicking relative activities of different conditions. Data are presented as mean ± SD (n =3 independent experiments).


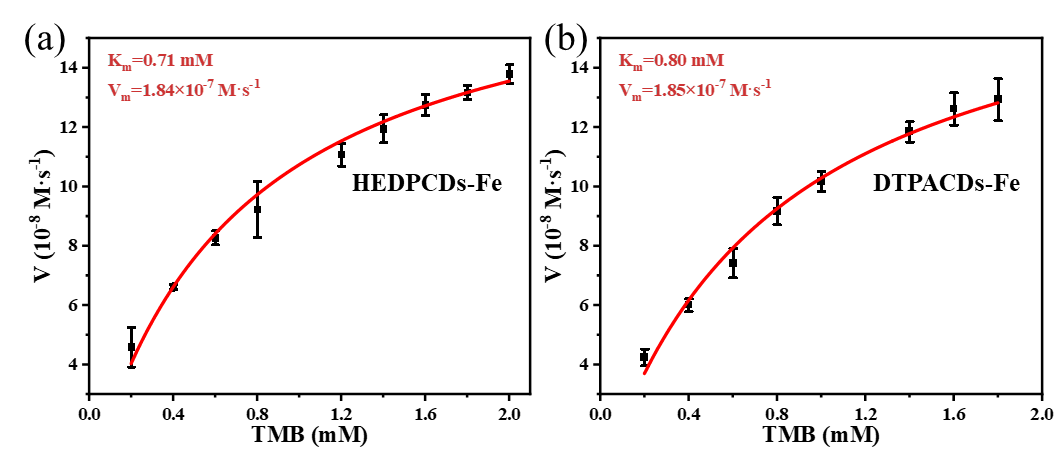


**Figure S9.** OXD-mimicking steady-state kinetic analysis for TMB substrate of (a) HEDPCDs-Fe and (b) DTPACDs-Fe. Data are presented as mean ± SD (n =3 independent experiments).

**Figure S10.** The EPR spectra of DMPO, H_2_O_2_, HEDPCDs-Fe + H_2_O_2,_ and HEDPCDs-Fe + H_2_O_2_ + UV.

**Table S1.** Comparison of the POD-mimicking catalytic activity of CDs-Fe with other reported nanozymes.

| Material | Strategy | Substrates | K_m_ (mM) | V_m_ (10^-8^ Μ s^-1^) | Complexity | Applicability | Reference |
| --- | --- | --- | --- | --- | --- | --- | --- |
| CDs@Cu_4_O_3_ | Dopping | OPD | 7.96 | 35.95 | Medium | Biotechnology | ^[3]^ |
| PEI@Mn/Cu^+^-CDs(solid) | Post-modification | TMB | 0.27 | 10.54 | High | Drug-resistant bacteria | ^[4]^ |
|  |  | H_2_O_2_ | 0.658 | 17.60 |  |  |  |
| SA Ir-CDs | Dopping | TMB | 0.25 | 11.81 | Medium | Detection Hg^2+^ | ^[5]^ |
|  |  | H_2_O_2_ | 0.63 | 1.05 |  |  |  |
| Co_9_S_8_/C | Heterojunctions | TMB | 0.64 | 10.23 | Medium | Detection of ascorbic acid | ^[6]^ |
|  |  | H_2_O_2_ | 7.91 | 6.05 |  |  |  |
| S/Cu-NC | Co-doppong | TMB | 0.19 | 4.55 | High | Detection melatonin | ^[7]^ |
|  |  | H_2_O_2_ | 0.75 | 3.26 |  |  |  |
| TPPCDs-Fe | Ligand-regulation | TMB | 1.17 | 4.54 | Low | antioxidant | This work |
|  |  | H_2_O_2_ | 0.25 | 3.37 |  |  |  |
| HEDPCDs-Fe | Ligand-regulation | TMB | 5.23 | 26.84 | Low | antioxidant | This work |
|  |  | H_2_O_2_ | 0.085 | 6.74 |  |  |  |
| DTPACDs-Fe | Ligand-regulation | TMB | 1.32 | 8.10 | Low | antioxidant | This work |
|  |  | H_2_O_2_ | 0.32 | 3.65 |  |  |  |

**Table S2.** Comparison of the OXD-mimicking catalytic activity of CDs-Fe with other reported nanozymes.

| Material | Strategy | Substrates | K_m_ (mM) | V_m_ (10^-8^ Μ s^-1^) | Complexity | Applicability | Reference |
| --- | --- | --- | --- | --- | --- | --- | --- |
| CDs@Cu_4_O_3_ | Dopping | OPD | 9.13 | 13.70 | Medium | Biotechnology | ^[3]^ |
| PEI@Mn-CDs | Post-modification | TMB | - | - | Medium | Drug-resistant bacteria | ^[4]^ |
| Co_9_S_8_/C | Heterojunctions | TMB | 0.123 | 2.38 | Medium | Detection of ascorbic acid | ^[6]^ |
| Fe-Se/NC | Co-doppong | TMB | 0.10 | 99.6 | High | Detection of SCCs | ^[8]^ |
| TPPCDs-Fe | Ligand-regulation | TMB | 0.73 | 22.60 | Low | antioxidant | This work |
| HEDPCDs-Fe | Ligand-regulation | TMB | 0.71 | 18.40 | Low | antioxidant | This work |
| DTPACDs-Fe | Ligand-regulation | TMB | 0.80 | 18.50 | Low | antioxidant | This work |

**Table S3.** The catalytic constant *k*_cat_ and catalytic efficiency *k*_cat_/*K*_m_ values for TMB and H_2_O_2_ of TPPCDs-Fe, HEDPCDs-Fe, and DTPACDs-Fe.

| Nanozyme name | Substrates | Catalytic type | E (mM) | *k*_cat_ (×10^-3^ s^-1^) | *k*_cat_/*K*_m_ (M^-1^ s^-1^) |
| --- | --- | --- | --- | --- | --- |
| TPPCDs-Fe | TMB | POD | 0.402 | 0.11 | 0.066 |
|  | H_2_O_2_ | POD |  | 0.084 | 0.34 |
|  | TMB | OXD |  | 0.56 | 0.77 |
|  | TMB | POD + OXD |  | 1.02 | 1.20 |
| HEDPCDs-Fe | TMB | POD | 0.708 | 0.38 | 0.072 |
|  | H_2_O_2_ | POD |  | 0.095 | 1.12 |
|  | TMB | OXD |  | 0.26 | 0.37 |
|  | TMB | POD + OXD |  | 0.73 | 0.70 |
| DTPACDs-Fe | TMB | POD | 0.454 | 0.18 | 0.14 |
|  | H_2_O_2_ | POD |  | 0.080 | 0.25 |
|  | TMB | OXD |  | 0.41 | 0.51 |

**Table S4**. Summary of Core Structure-Activity Relationships

| Nanozyme | Ligand Key Features | Kinetic Highlights | Dominant Advantage |
| --- | --- | --- | --- |
| TPPCDs-Fe | Conjugated aromatics + N/P coordination | High OXD activity, strong synergism | UV-induced catalysis |
| HEDPCDs-Fe | Compact O-donor phosphonate | Lowest K_m_ (H_2_O_2_), highest Vm(POD) | Peroxidase-mimicking rate |
| DTPACDs-Fe | Rigid N/O hexadentate chelate | Balanced K_m_ (TMB/ H_2_O_2_), moderate efficiency | Affinity-driven POD activity |

**References**

[1] X. Li, Y. Li, M. Du, Y. V. Petrov, V. E. Baulin, Y. Wang, H. Yuan, Y. Zhou, B. Li, Target-Oriented Synthesis of Triphenylphosphine Functionalized Carbon Dots with Negative Charge for ROS Scavenging and Mitochondrial Targeting. *ACS Applied Materials & Interfaces* **2024,** *16*, 28991.

[2] J. Dong, G. Liu, Y. V. Petrov, Y. Feng, D. Jia, V. E. Baulin, A. Y. Tsivadze, Y. Zhou, B. Li, EDTA-Functionalized Carbon Dots–Metal Nanozymes Based on Coordination Chemistry: Species of Metal Ions Determines Enzyme-Mimicking Catalyzed Activities. *ACS Materials Letters* **2024,** *6*, 1112.

[3] F. Li, Q. Chang, N. Li, C. Xue, H. Liu, J. Yang, S. Hu, H. Wang, Carbon dots-stabilized Cu4O3 for a multi-responsive nanozyme with exceptionally high activity. *Chemical Engineering Journal* **2020,** *394*.

[4] Q. Duan, L. Tang, S. Xu, D. Yang, Y. Xu, Metal ions-driven multi-enzyme activity of PEI-carbon dots-based nanozyme to improve antibacterial ability. *Chemical Engineering Journal* **2025,** *511*, 161957.

[5] T. Li, J. Xia, M. Wu, C. Liu, Y. Sun, W. Zhao, M. Qian, W. Wang, W. Duan, S. Xu, Single-Atom Iridium-doped Carbon Dots Nanozyme with High Peroxidase-Like Activity as Colorimetric Sensors for Multimodal Detection of Mercury Ions. *Small* **2025,** *21*, 2408785.

[6] S. Li, E. Pang, N. Li, Q. Chang, J. Yang, S. Hu, A bifunctional nanozyme of carbon dots-mediated Co9S8 formation. *Journal of Colloid and Interface Science* **2022,** *608*, 1348.

[7] C. Zhou, N. Wang, Y. Su, J. Liu, Y. Lv, X. Su, Sulfur/nitrogen co-doped carbon-based copper nanozyme with high peroxidase-like activity for dual-channel paper-based detection of melatonin. *Sensors and Actuators B: Chemical* **2024,** *418*, 136246.

[8] E. Ren, H. Qiu, Z. Yu, M. Cao, M. Sohail, G. Lu, X. Zhang, Y. Lin, Nanozyme sensor array based on Fe, Se co-doped carbon material for the discrimination of Sulfur-containing compounds. *Journal of Hazardous Materials* **2024,** *470*, 134127.
